# Minding the Gap: Follow-Up Cardiovascular Care After Clonal Hematopoiesis Detection

**DOI:** 10.1016/j.jacadv.2026.103357

**Published:** 2026-10-16

**Authors:** Ohad Oren, Abhay Singh, Hetty E. Carraway, Venu Menon

**Affiliations:** a Department of Cardiovascular Medicine, Heart, Vascular, and Thoracic Institute, Cleveland Clinic Foundation, Cleveland, Ohio, USA; b Leukemia Program, Taussig Cancer Institute, Cleveland Clinic, Cleveland, Ohio, USA

**Keywords:** cardio-oncology, cardiovascular prevention, cardiovascular referral, cardiovascular risk, CCUS, CHIP, clonal hematopoiesis

Each year, hundreds of thousands of patients undergo genomic sequencing of blood or bone marrow samples during the evaluation of unexplained cytopenias; suspected hematologic malignancies; or as part of cancer diagnosis, treatment, and survivorship care.^1^ Across these clinical settings, approximately 10% to 30% of patients are found to harbor somatic mutations in hematopoietic stem and progenitor cells, many involving pathogenic variants associated with an increased risk of cardiovascular disease and/or increased risk for hematological malignancy.^2^ When these mutations are detected at a variant allele frequency (VAF) of at least 2%, a threshold used to define the proportion of sequencing reads containing the variant allele, they are categorized as clonal hematopoiesis of indeterminate potential (CHIP) in the absence of unexplained cytopenias or hematologic malignancy, or as clonal cytopenia of undetermined significance (CCUS) when persistent unexplained cytopenias are present.^2^ Although these findings are generally managed by hematologists, a growing body of evidence has established that many of these pathogenic variants also carry important cardiovascular implications, including significantly elevated risks of atherosclerotic cardiovascular disease, heart failure, arrhythmia, and venous thrombosis.^3^ Many of these patients continue to remain exclusively under the care of our hematology and oncology colleagues, representing a major missed opportunity for cardiovascular risk assessment and prevention. To address this gap, we propose a practical framework for cardiovascular referral of patients with CHIP/CCUS detected during routine hematologic and oncologic care (Figure 1).

**Figure 1:**
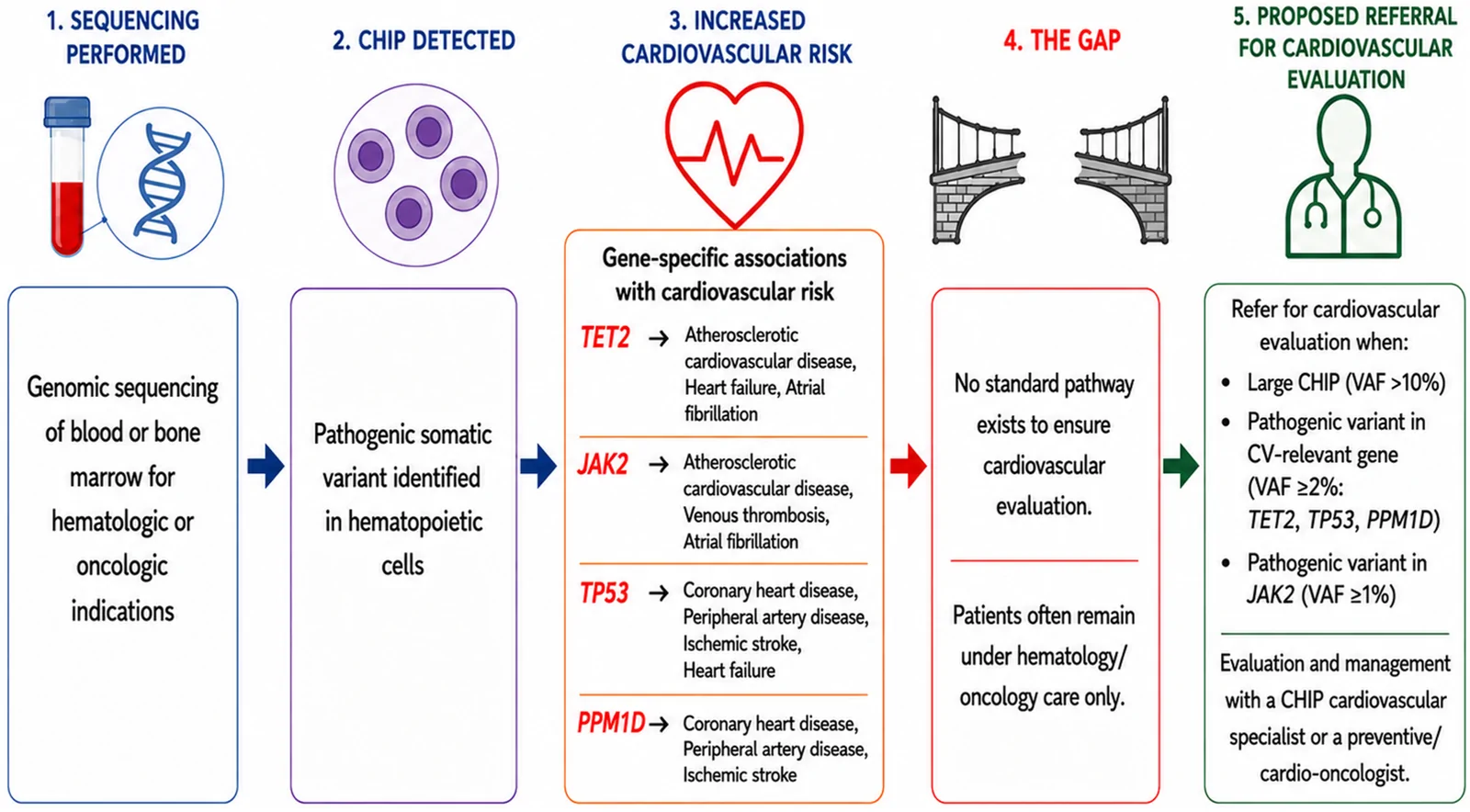
Proposed Clinical Pathway for Cardiovascular Referral After Detection of Clonal Hematopoiesis of Indeterminate Potential (CHIP)/Clonal Cytopenia of Undetermined Significance (CCUS) Genomic sequencing performed for hematologic or oncologic indications identifies patients with CHIP/CCUS, some of whom harbor somatic mutations associated with increased cardiovascular risk. A practical referral pathway is outlined for high-risk patients, including those with large CHIP/CCUS (VAF >10%), CHIP/CCUS with *TET2*, *TP53*, or *PPM1D* mutations (VAF ≥2%), or *JAK2* mutations (VAF ≥1%). CCUS = clonal cytopenia of undetermined significance; CHIP = clonal hematopoiesis of indeterminate potential; VAF = variant allele frequency.

CHIP and CCUS represent acquired processes in which hematopoietic stem cells and precursors accumulate mutations through repeated replication cycles, accelerated by mutagenic exposures (ie, tobacco, cytotoxic chemotherapy, radiation, etc) and germline vulnerabilities in DNA repair pathways.^4^ Pathogenic variants in genes governing DNA methylation (ie, *DNMT3A*, *TET2*), signal transduction (ie, *JAK2*), epigenetic regulation (ie, *ASXL1*), and RNA splicing (ie, *SF3B1*, *SRSF2*) may confer selective advantages to mutant hematopoietic stem cells, culminating in the generation of expanded clones with distinct, gene-specific biologic properties, including myeloid lineage bias, proinflammatory reprogramming of myeloid effector cells, and enhanced platelet activation. Because somatic mutations are acquired over the lifespan through repeated hematopoietic stem cell replication, the prevalence of CHIP/CCUS is strongly age-dependent: it is rare before age 40, detectable in approximately 10% of individuals over 70 years of age, and approaches 20% among nonagenarians.^2^ However, with sufficiently sensitive deep sequencing, small clonal populations may be nearly ubiquitous with aging.^5^ Driven by population aging, broader adoption of next-generation sequencing , and increasingly sensitive sequencing platforms capable of detecting clones with variant allele frequencies as low as 0.1%, the number of patients identified with somatic mutations in leukemia-associated genes is expected to rise substantially over the coming years.

The clinical consequences of CHIP/CCUS are diverse. First, they predispose to the development of myeloid and lymphoid malignancies at a rate of 0.5% to 1.0% per year.^2^, ^6^ Second, they substantially increase the risk of cardiovascular disease, which accounts for a significant proportion of the excess mortality observed in these populations.^7^ The cardiovascular implications of CHIP are gene-specific with *TET2* and *JAK2* variants exhibiting the strongest and most consistent associations with future manifest atherosclerotic cardiovascular disease, with HRs of 1.9 and up to 12-fold, respectively.^2^, ^7^ In contrast, mutations in DNA damage repair genes (*TP53* and *PPM1D*) have been associated with a broader spectrum of adverse cardiovascular outcomes, including coronary heart disease, incident peripheral artery disease, and markedly increased all-cause mortality (HR: 2.78).^7^ Notably, the excess cardiovascular risk associated with CHIP, particularly high-risk genotypes such as *TET2*, *JAK2*, and *TP53*, is of a magnitude comparable to or greater than that of traditional cardiovascular risk factors, including type 2 diabetes and hypertension. Several expert groups have accordingly proposed that CHIP be considered a risk-enhancing factor, warranting aggressive optimization of modifiable risk factors, including lipids, blood pressure, and glycemic index, as well as nicotine avoidance and weight reduction, through both lifestyle interventions and pharmacotherapy.^8^ A 2026 American Heart Association Scientific Statement on CHIP and cardiovascular disease further endorsed individualized cardiovascular risk assessment and primary and secondary prevention in the management of patients with CHIP/CCUS.^9^ Building on the growing evidence linking CHIP to cardiovascular disease, we propose and have adopted a multidisciplinary referral pathway to standardize cardiovascular evaluation after the detection of CHIP/CCUS. The immediate priority is to ensure that patients harboring high-risk somatic mutations receive comprehensive cardiovascular risk assessment and management of conventional cardiovascular risk factors and consideration of enrollment in clinical trials while mutation-specific therapies await validation in prospective clinical trials.

Our structured referral framework is presented in Figure 1. When whole-genome sequencing or targeted sequencing of blood or bone marrow specimens identifies pathogenic variants in cardiovascular-relevant genes (eg, *TET2*, *TP53*, *PPM1D*) at a VAF ≥2% (or ≥1% for *JAK2*) or large CHIP/CCUS (VAF>10%), the managing hematologist or oncologist should consider referral for cardiovascular evaluation. Ideally, patients should be evaluated by a dedicated CHIP cardiovascular specialist, where available, or alternatively by a cardio-oncologist or preventive cardiologist with expertise in clonal hematopoiesis. Close collaboration between hematology/oncology and cardiovascular medicine is essential to ensure that cardiovascular evaluation is tailored to the patient's hematologic and oncologic context, including the underlying diagnosis, distinction between somatic and germline variants when relevant, and ongoing or planned cardiotoxic therapies that may influence cardiovascular management or necessitate coordinated clinical decision-making.

The cardiovascular evaluation should follow previously proposed practical approaches for the management of patients with CHIP/CCUS and include a comprehensive assessment of traditional cardiovascular risk factors,^8^, ^9^ including smoking status, family history of premature atherosclerotic cardiovascular disease, body mass index, blood pressure, lipid profile, and hemoglobin A_1c_. Existing imaging obtained as part of oncologic care should also be reviewed opportunistically for findings that may refine cardiovascular risk assessment, such as coronary artery calcification on chest computed tomography or breast arterial calcifications on mammogram. Echocardiography, including assessment of global longitudinal strain when appropriate, should be considered in patients with heart failure symptoms or those anticipated to receive potentially cardiotoxic therapies. In the absence of evidence, we do not advocate routine rhythm monitoring solely based on the presence of CHIP. In selected patients, however, a lower threshold for ambulatory monitoring may be reasonable, particularly with *TET2* or *JAK2* mutations given their associations with atrial fibrillation. Management should emphasize individualized, mutation-informed intensification of primary and secondary cardiovascular prevention in accordance with contemporary practical guidance, within a multidisciplinary care model involving hematology, oncology (when applicable), genetics, and cardiovascular medicine.^8^, ^9^

It is important to note that no prospective study has demonstrated that identification of CHIP followed by implementation of cardiovascular risk reduction strategies improves clinical outcomes compared with standard care. The proposed framework is therefore based on biological plausibility, observational evidence, and extrapolation from established cardiovascular prevention principles rather than prospective or randomized trial data. In addition, the optimal frequency of cardiovascular assessments and the cost-effectiveness of systematic referral remain undefined. This also underscores the importance of considering enrollment in available clinical trials and educating patients about such opportunities.

The alternative is difficult to justify: patients with mutations associated with substantial cardiovascular risk are currently identified without any standardized pathway for cardiovascular evaluation. The convergence of expanding next-generation sequencing utilization, a growing evidence base linking specific mutations to cardiovascular risk, and a broadening cardiometabolic therapeutic toolkit creates both the opportunity and the obligation to bridge the gap between hematologic detection and cardiovascular evaluation and management. Prospective registries and pragmatic trials evaluating the impact of structured cardiovascular referral pathways on clinical outcomes in patients with CHIP/CCUS are needed. In the interim, the framework proposed here offers a practical starting point for ensuring that the cardiovascular implications of CHIP/CCUS are no longer overlooked.

## Funding support and author disclosures

The authors have reported that they have no relationships relevant to the contents of this paper to disclose.
